# 24-Hour movement behaviour and quality of life among Chinese caregivers of children with intellectual disabilities: a compositional analysis

**DOI:** 10.1016/j.pmedr.2026.103622

**Published:** 2026-09-06

**Authors:** Yanan Liu, Yangchen Liu, Jianing Ding, Yaqing Yuan, Glenn Roswal

**Affiliations:** a Department of Physical Education, Shandong Jianzhu University, Jinan, Shandong 250101, China; b School of Law, Shandong Jianzhu University, Jinan, Shandong 250101, China; c Department of Physical Education, Fujian Agriculture and Forestry University, Fuzhou, Fujian 350002, China; d College of Sports and Health, Shandong Sport University, Jinan, Shandong 250102, China; e School of Health Professions and Wellness, Jacksonville State University, Jacksonville, AL 36265, USA

**Keywords:** Intellectual disability, Caregivers, Quality of life, Sedentary behaviour, Physical activity, Compositional data analysis

## Abstract

**Objective:**

To examine association between 24-h movement behaviour and quality of life (QoL) among caregivers of children with intellectual disabilities and to estimate QoL differences under hypothetical time reallocations.

**Methods:**

This cross-sectional study included 225 caregivers in Jinan, China, from October 2025 to January 2026 (mean age, 39.40 ± 8.66 years; 81.78% female). Movement behaviours were measured using ActiGraph accelerometers, and QoL was assessed using the World Health Organization Quality of Life-Brief Version (WHOQOL-BREF). Compositional analysis was used to describe movement behaviour, estimate associations using pivot isometric log-ratio regression, and model 10- and 30-min reallocations among sleep, sedentary behaviour, light physical activity and moderate-to-vigorous physical activity.

**Results:**

Caregivers spent 411.03 min/day sleeping, 624.84 min/day sedentary, 357.29 min/day in light physical activity, and 46.84 min/day in moderate-to-vigorous physical activity. The overall WHOQOL-BREF score was 49.66. Sedentary behaviour was associated with lower QoL (γ = −7.63; 95% *CI*, −13.65, −1.60), whereas moderate-to-vigorous physical activity was associated with higher QoL (γ = 8.26; 95% *CI*, 6.07, 10.46). Reallocating 30 min/day from sedentary behaviour to moderate-to-vigorous physical activity predicted a 3.87-point higher score.

**Conclusions:**

Caregivers' 24-h movement behaviour was associated with QoL. These findings support further investigation of strategies to reduce avoidable sedentary time and facilitate physical activity.

## Highlights

• 24-Hour movement behaviours were examined in caregivers of Chinese children with intellectual disabilities.
• Sedentary behaviour was associated with poorer caregiver quality of life.
• Moderate-to-vigorous physical activity showed the strongest favourable association with quality of life.
• Replacing sedentary time with moderate-to-vigorous physical activity predicted higher quality-of-life scores.
• Findings support movement-based health promotion for caregivers of children with intellectual disabilities.

## 1 Introduction

Quality of life (QoL) is commonly conceptualised as a multidimensional construct that reflects individuals' perceptions of their physical health, psychological wellbeing, social relationships, and environmental circumstances (Skevington et al., 2004; The WHOQOL Group, 1995). Within this context, QoL is a particularly important outcome for caregivers of children with intellectual disabilities, because caregiving demands and family circumstances may profoundly shape caregivers' own health, wellbeing and daily functioning. More broadly, raising a child with intellectual disabilities may have a substantial impact not only on primary caregivers but also on siblings and the family system as a whole (Trute and Hiebert-Murphy, 2002). Caregivers of children with intellectual disabilities often face distinctive caregiving, practical, and emotional challenges related to the pervasive and long-term support needs of their children (Alnahdi and Schwab, 2024; Lahaije et al., 2024b). In daily life, they may need to coordinate education, rehabilitation, medical care, transport, behavioural support, and daily living assistance while also maintaining employment, family responsibilities, and their own wellbeing. As these responsibilities may continue across different stages of family life, caregiving can become intensive and sustained (Tadema and Vlaskamp, 2010), sometimes exceeding caregivers' perceived capacities and restricting their social participation (Schmidt et al., 2017). Such sustained demands may increase the risk of stress, anxiety, and depressive symptoms among primary caregivers (Miodrag and Hodapp, 2010; Scherer et al., 2019). They may also reduce the time and energy available for self-care and health-promoting activities, thereby contributing to poorer physical and emotional health, lower QoL, and greater caregiving burden . Previous studies have shown that caregiver wellbeing and family QoL in families of children with intellectual disabilities are associated with multiple psychosocial and contextual factors, including emotional support, family resources, child support needs, disability severity, employment, social support, psychological distress, and family resilience (Barratt et al., 2025; Lahaije et al., 2024a; Luitwieler et al., 2021; Vanderkerken et al., 2019; Yildirim et al., 2026). These findings highlight the complex psychosocial and contextual conditions under which caregivers of children with intellectual disabilities maintain their own wellbeing. However, less attention has been given to modifiable behavioural pathways through which caregiving demands may influence caregivers' QoL. In particular, the way daily time is distributed across sleep, sedentary behaviour, and physical activity may offer an important time-use perspective, because caregiving demands can shape not only caregivers' psychological resources and social participation, but also the structure of their everyday routines.

The 24-h movement behaviours provide a useful framework for examining this pathway. A full day is composed of sleep, sedentary behaviour, light physical activity, and moderate-to-vigorous physical activity, each of which may have implications for health and wellbeing. Evidence from 24-h movement behaviours research suggests that daily movement behaviour composition is associated with health-related QoL and mental health in different populations. For example, compositional studies among preschool and primary school children have reported that more favourable distributions of sleep, sedentary behaviour, and physical activity are related to better health-related QoL, and that reallocating time to moderate-to-vigorous physical activity or sleep may be associated with higher scores (Nyam et al., 2025; Zhang et al., 2025). Evidence from adult occupational settings also suggests that reallocating sedentary or light-intensity time to sleep may be associated with lower psychological distress and higher work engagement (Kitano et al., 2020). Although these findings come from populations other than caregivers of children with intellectual disabilities, they suggest that daily movement behaviour composition may be relevant for understanding wellbeing in groups whose daily routines are shaped by sustained demands and limited discretionary time. This perspective is especially meaningful for caregivers of children with intellectual disabilities, because caregiving may reshape the entire daily time budget. Caregivers may experience shortened or fragmented sleep due to night-time care or worry, prolonged sedentary time related to supervision, transport, appointments, documentation, or waiting during services and fewer protected opportunities for health-promoting physical activity. At the same time, light physical activity may be embedded in household labour and hands-on care, whereas moderate-to-vigorous physical activity may require intentional time, respite, physical capacity, and family support. Thus, daily movement behaviour in this population is not merely an individual lifestyle preference, but may reflect the distribution of care, autonomy, fatigue, support, and opportunity across the family day.

Despite the relevance of this time-use perspective, little is known about how caregivers' 24-h movement behaviour composition relates to their own QoL. Much existing evidence on movement behaviours and wellbeing has examined sleep, sedentary behaviour or physical activity separately. This approach is limited because time use is finite and inherently co-dependent: increasing time in one movement behaviour necessarily reduces time available for at least one other movement behaviour (Chastin et al., 2015; Pedišić, 2014). Conventional regression models that treat raw minutes as independent exposures may therefore obscure the time-use substitution being estimated. Compositional data analysis addresses this problem by treating 24-h movement behaviours as relative parts of a whole and using log-ratio methods that respect the closed structure of time-use data (Dumuid et al., 2020; Dumuid et al., 2019). In addition, compositional isotemporal substitution can estimate predicted differences in QoL when time is hypothetically reallocated between movement behaviours, such as replacing sedentary behaviour with light or moderate-to-vigorous physical activity. Accordingly, the present study applied a compositional approach to examine caregivers' 24-h movement behaviour composition and QoL in families of children with intellectual disabilities. Specifically, this study aimed to describe caregivers' 24-h movement behaviour composition and QoL profiles, examine how the relative distribution of sleep, sedentary behaviour, light physical activity, and moderate-to-vigorous physical activity was associated with QoL, and estimate predicted differences in QoL under hypothetical time reallocations between these movement behaviours.

## 2 Methods

### 2.1 Study design and participants

This cross-sectional study was conducted in Jinan, Shandong Province, China, between October 2025 and January 2026. A total of 247 parents or primary caregivers of children with intellectual disabilities were recruited through special education schools. Participants were eligible if they were the main caregiver of a school-aged child with documented intellectual disability. Of the 247 caregivers recruited, 13 were excluded because of insufficient accelerometer data to construct a valid 24-h movement behaviour composition, and nine were excluded because of incomplete the World Health Organization Quality of Life-Brief Version (WHOQOL-BREF) data. The final analytic sample therefore comprised 225 parents or primary caregivers. Caregiver and family characteristics collected included caregiver age, sex, relationship to the child, education, employment status, household income, daily caregiving hours, child age, and intellectual disabilities severity. Daily caregiving hours were assessed by asking caregivers to report the average number of hours per day they personally spent providing care for the child.

This study was conducted in accordance with the Declaration of Helsinki and was approved by the Ethics Committee of Shandong Jianzhu University (approval number: H20051S-2401). All participants were parents or primary caregivers of children with intellectual disabilities. Written informed consent to participate in this study was obtained from all participants themselves.

### 2.2 Measures

#### 2.2.1 24-Hour movement behaviours

Caregivers' 24-h movement behaviours were objectively assessed using ActiGraph wGT3X-BT triaxial accelerometers (ActiGraph, Pensacola, FL, USA). The devices were programmed to record raw acceleration at 30 Hz, and the data were subsequently reintegrated into 15-s epochs with the normal filter in ActiLife software, version 6.13.4. Participants were asked to wear the accelerometer on the right hip, close to the iliac crest, for seven consecutive days, including five weekdays and two weekend days. They were instructed to remove the device only during water-based activities, such as bathing or swimming, to ensure continuous recording across both sleep and waking periods.

Accelerometer files were downloaded and processed in ActiLife. Periods classified as non-wear using the Choi algorithm (Choi et al., 2011) were treated as unobserved time and excluded before behaviour-specific durations were calculated; no behaviour-specific imputation was performed. As participants were instructed to remove the device only for bathing, swimming, or other water-based activities, removal periods that met the Choi non-wear criteria were handled in the same manner. A valid day was defined as at least 480 min of waking wear time and participants were included in the analysis if they provided at least three valid days, including at least two weekdays and one weekend day. Sleep duration was estimated using an accelerometer-based bedrest identification approach validated for adults wearing waist-worn accelerometers (Tracy et al., 2018). During waking hours, sedentary behaviour was defined as <200 vector magnitude counts per minute, light physical activity as 200–2689 vector magnitude counts per minute, and moderate-to-vigorous physical activity as ≥2690 vector magnitude counts per minute according to the Sasaki et al. thresholds (Sasaki et al., 2011). For each participant, the daily durations of sleep, sedentary behaviour, light physical activity, and moderate-to-vigorous physical activity were first averaged across all valid days, with each valid day weighted equally. The resulting participant-level four-part composition was then proportionally rescaled so that the four behaviours summed to 1440 min. This proportional closure applied the same scaling factor to all four behaviours, thereby preserving their relative distribution.

#### 2.2.2 Quality of life

Caregivers' QoL was assessed using WHOQOL-BREF domain scores. The domains comprise physical health, psychological wellbeing, social relationships and environment, with higher scores indicating better perceived QoL (Skevington et al., 2004; The WHOQOL Group, 1998). Scores were transformed to a 0–100 metric. The overall QoL score was calculated as the arithmetic mean of the four transformed domain scores, consistent with the approach used in previous research (Doroszkiewicz et al., 2021; Dumitrescu et al., 2026), and was used as the primary outcome because the study focused on caregivers' global perceived life circumstances. This derived score was treated as a study-specific summary measure rather than an official WHOQOL-BREF index. The WHOQOL-BREF has been widely used to assess QoL among parents and caregivers of children with disabilities, including caregivers of children with intellectual disabilities, and previous studies have supported its applicability for capturing caregivers' physical, psychological, social, and environmental life conditions (Ali et al., 2021; Lin et al., 2009). Psychometric evidence from caregiver populations also suggests that the WHOQOL-BREF is a generally reliable and valid instrument for assessing adult caregivers' QoL, although performance may vary across domains and cultural contexts . In the present study, the internal consistency coefficient for the overall WHOQOL-BREF score was 0.74, indicating acceptable reliability.

### 2.3 Statistical analysis

Analyses followed standard compositional data analysis procedures for time-use epidemiology (Chastin et al., 2015; Dumuid et al., 2020; Dumuid et al., 2019). Descriptive statistics were calculated for demographic variables, movement behaviours and WHOQOL-BREF scores. For the movement behaviour composition, arithmetic means and standard deviations were reported for interpretability, and closed geometric means were used to describe the compositional center. A log-ratio variation matrix was calculated to assess the pairwise proportional co-variation between movement behaviours. Smaller values indicate stronger co-dependence between two movement behaviours, whereas larger values indicate greater relative dispersion.

Because the four movement behaviours sum to 1440 min, raw minutes were not entered directly as independent predictors. Instead, isometric log-ratio coordinates were calculated. Using a pivot-coordinate approach, the same composition was re-expressed under four alternative coordinate representations, with each movement behaviour occupying the first pivot coordinate in turn. The first pivot coordinate represents the relative share of the target movement behaviour compared with the geometric mean of the remaining movement behaviours. A positive coefficient for this coordinate indicates that a higher relative share of that movement behaviour is associated with higher QoL, after adjustment for covariates and the remaining composition.

Adjusted linear regression models estimated the association between movement behaviour composition and overall WHOQOL-BREF score. Models adjusted for caregiver age, sex, relationship to the child, education, employment status, household income, daily caregiving hours, child age, and severity of intellectual disability. The covariates were selected based on previous literature and their potential associations with both caregivers' movement behaviours and QoL, covering caregiver sociodemographic characteristics, caregiving demands, and child characteristics. Compositional isotemporal substitution used the mean observed composition as the reference and created hypothetical new compositions by reallocating 10 or 30 min from one movement behaviour to another while holding the total time constant at 1440 min. Predicted differences in overall QoL and 95% confidence intervals (CI) were calculated from the fitted model and its variance-covariance matrix. Analyses were conducted using R, version 4.6.1 (R Foundation for Statistical Computing, Vienna, Austria).

## 3 Results

### 3.1 Participant characteristics

The mean caregiver age was 39.40 ± 8.66 years. Of the caregivers, 184 (81.78%) were female, and 41 (18.12%) were male. Mothers represented the largest caregiver group. Children had a mean age of 9.13 ± 2.33 years, and severity of intellectual disabilities was distributed across moderate and severe categories. Mean daily caregiving time was 5.51 ± 1.66 h (Table 1).

**Table 1:** Characteristics of caregivers of children with intellectual disabilities and their children in Jinan, China, October 2025–January 2026.

Characteristic	Category/statistic	Value
Caregiver age	Mean ± SD	39.40 ± 8.66
Caregiver sex	Female/Male	184 (81.78%)/41 (18.12%)
Relationship	Mother/Father/Grandparent/Other	167 (74.22%)/36 (16.00%)/19 (8.44%)/3 (1.33%)
Education	Junior/High school/College/Bachelor+	45 (20.00%)/79 (35.11%)/62 (27.56%)/39 (17.33%)
Employment	Full-time/Part-time/Flexible/Unemployed	87 (38.67%)/29 (12.89%)/51 (22.67%)/58 (25.78%)
Household income	<5000/5000–9999/≥10,000 Chinese yuan	96 (42.67%)/86 (38.22%)/43 (19.11%)
Child age	Mean ± SD	9.13 ± 2.33
Intellectual disability severity	Moderate/Severe	130 (57.78%)/95 (42.22%)
Daily caregiving time	Mean ± SD	5.51 ± 1.66

Abbreviation: SD, standard deviation.

### 3.2 24-Hour movement behaviours composition and quality-of-life profile

Caregivers spent 411.03 min/day sleeping, 624.84 min/day sedentary, 357.29 min/day in light physical activity, and 46.84 min/day in moderate-to-vigorous physical activity. These values corresponded to 28.54%, 43.39%, 24.81% and 3.25% of the 24-h day, respectively.

The mean overall WHOQOL-BREF score was 49.66 ± 8.34. Domain scores were similar in magnitude: physical health 49.15 ± 10.64, psychological wellbeing 48.10 ± 9.12, social relationships 50.26 ± 9.27, and environment 51.13 ± 11.64. This profile suggested that perceived QoL was not confined to one domain but was distributed across physical, psychological, social, and environmental dimensions (Table 2).

**Table 2:** Descriptive statistics for 24-h movement behaviours and quality-of-life scores among caregivers of children with intellectual disabilities in Jinan, China, October 2025–January 2026.

Variable	Mean ± SD	Closed geometric mean
Sleep, min/day	407.03 ± 57.22	411.03
Sedentary behaviour, min/day	620.70 ± 72.03	624.84
Light physical activity, min/day	351.92 ± 61.14	357.29
Moderate-to-vigorous physical activity, min/day	41.59 ± 21.30	46.84
WHOQOL physical domain	49.15 ± 10.64	–
WHOQOL psychological domain	48.10 ± 9.12	–
WHOQOL social relationships domain	50.26 ± 9.27	–
WHOQOL environment domain	51.13 ± 11.64	–
WHOQOL overall score	49.66 ± 8.34	–

Abbreviation: SD, standard deviation.

### 3.3 Log-ratio variation between movement behaviours

The variation matrix indicated the strongest proportional co-dependence between sleep and sedentary behaviour (0.05). The largest pairwise variation was observed between light physical activity and moderate-to-vigorous physical activity (0.32), suggesting greater relative dispersion between these components (Table 3).

**Table 3:** Log-ratio variation matrix for the 24-h movement behaviour composition among caregivers of children with intellectual disabilities in Jinan, China, October 2025–January 2026.

Movement behaviour	Sleep	Sedentary behaviour	Light physical activity	Moderate-to-vigorous physical activity
Sleep	0.00	0.05	0.06	0.30
Sedentary behaviour	0.05	0.00	0.07	0.29
Light physical activity	0.06	0.07	0.00	0.32
Moderate-to-vigorous physical activity	0.30	0.29	0.32	0.00

### 3.4 Compositional regression

The adjusted compositional regression model identified two clear associations with overall QoL (Table 4). A higher relative share of sedentary behaviour was associated with a lower WHOQOL-BREF overall score (γ = −7.63; 95% *CI*, −13.65, −1.60). By contrast, a higher relative share of moderate-to-vigorous physical activity was associated with a higher overall score (γ = 8.26; 95% *CI*, 6.07, 10.46). Sleep and light physical activity were not statistically clear correlates of overall QoL after covariate adjustment.

**Table 4:** Adjusted associations between 24-h movement behaviour composition and overall quality of life among caregivers of children with intellectual disabilities in Jinan, China, October 2025–January 2026.

Movement behaviour	γ	95% *CI*
Sleep	−0.11	−5.84, 5.62
Sedentary behaviour	−7.63	−13.65, −1.60
Light physical activity	−0.52	−5.87, 4.82
Moderate-to-vigorous physical activity	8.26	6.07, 10.46

Abbreviation: CI, confidence interval.

Note: γ represents the first-pivot-coordinate coefficient when the named movement behaviour occupies the first coordinate. The four coefficients were obtained from alternative coordinate representations of the same adjusted compositional model.

### 3.5 Compositional isotemporal substitution

The isotemporal substitution analysis translated the regression pattern into time-reallocation scenarios (Table 5). Reallocating 10 min from sedentary behaviour to moderate-to-vigorous physical activity was associated with a 1.49-point higher predicted overall WHOQOL-BREF score, and reallocating 30 min was associated with a 3.87-point higher score. Reallocating time from sleep or light physical activity to moderate-to-vigorous physical activity also showed favourable predicted differences. In the opposite direction, reallocating 30 min from moderate-to-vigorous physical activity to sedentary behaviour was associated with a 7.63-point lower predicted score.

**Table 5:** Predicted differences in overall quality of life under hypothetical 10- and 30-min reallocations between 24-h movement behaviours among caregivers of children with intellectual disabilities in Jinan, China, October 2025–January 2026.

Reallocation	10-min difference (95% *CI*)	30-min difference (95% *CI*)
Sedentary behaviour -> Moderate-to-vigorous physical activity	1.49 (1.10, 1.88)	3.87 (2.85, 4.88)
Light physical activity -> Moderate-to-vigorous physical activity	1.40 (1.00, 1.80)	3.58 (2.52, 4.64)
Sleep -> Moderate-to-vigorous physical activity	1.39 (0.98, 1.79)	3.55 (2.49, 4.61)
Sedentary behaviour -> Sleep	0.10 (−0.08, 0.28)	0.32 (−0.22, 0.85)
Sleep -> Sedentary behaviour	−0.10 (−0.28, 0.08)	−0.30 (−0.85, 0.25)
Sedentary behaviour -> Light physical activity	0.09 (−0.09, 0.28)	0.29 (−0.26, 0.83)
Light physical activity -> Sedentary behaviour	−0.09 (−0.28, 0.09)	−0.27 (−0.83, 0.29)
Moderate-to-vigorous physical activity -> Sedentary behaviour	−1.82 (−2.30, −1.35)	−7.63 (−9.63, −5.63)

Abbreviation: CI, confidence interval.

## 4 Discussion

This study used a compositional isotemporal substitution framework to investigate the relationship between caregivers' 24-h movement behaviour composition and their own QoL in families of children with intellectual disabilities. The results revealed that caregivers spent a substantial proportion of their day in sedentary behaviour, averaging 624.84 min/day (43.39% of the 24-h period), while time in moderate-to-vigorous physical activity was limited, averaging 46.84 min/day (3.25% of the day). Caregivers' overall WHOQOL-BREF score was in the mid-range, and the four domain scores were broadly similar, suggesting that perceived QoL was not concentrated in a single domain but distributed across physical health, psychological wellbeing, social relationships, and environmental resources. This pattern is consistent with family QoL literature showing that caregiver wellbeing is shaped by multiple interacting conditions, including caregiving burden, material resources, emotional support, family functioning, service access, and child support needs (Alnahdi and Schwab, 2024; Luitwieler et al., 2021; Schmidt et al., 2017). The descriptive characteristics of the sample also reinforce this interpretation: most participants were mothers, and the mean daily caregiving time was 5.51 h, suggesting substantial daily caregiving involvement. Consequently, the observed movement behaviour composition should be understood within the context of sustained caregiving, rather than as a simple matter of lifestyle preference. Recent qualitative and quantitative evidence similarly suggests that caregivers caring for children with moderate to profound intellectual disabilities often experience reduced personal time, physical strain, psychological pressure, and restricted social participation (Barratt et al., 2025; Yildirim et al., 2026).

Sedentary behaviour showed the clearest adverse association with QoL. In the adjusted compositional regression model, a higher relative share of sedentary behaviour was associated with lower overall WHOQOL-BREF score. This finding is consistent with broader adult evidence linking sedentary behaviour with poorer health-related QoL and depressive symptoms (Boberska et al., 2018; del Pozo Cruz et al., 2020). However, in this caregiver group, sedentary behaviour time should not be interpreted narrowly as voluntary screen time or leisure sitting. It may include supervision, transport, waiting during rehabilitation or medical appointments, paperwork, school communication and passive recovery after physically or emotionally demanding care. The negative coefficient may therefore reflect more than the physiological consequences of prolonged sitting. It may also indicate a daily pattern marked by constrained autonomy, limited restorative opportunities and reduced access to self-directed activity. This interpretation also fits caregiver research showing that parental stress, child behavioural problems, inadequate support and chronic care demands are associated with poorer parental health and wellbeing (Hastings, 2002; Miodrag and Hodapp, 2010; Neece et al., 2012). For this reason, reducing sedentary behaviour time among these caregivers should not be framed only as a matter of individual motivation. It may depend on more practical supports, such as family assistance, access to temporary respite care, more flexible service scheduling, and opportunities for caregivers to move during otherwise sedentary caregiving routines.

In contrast, moderate-to-vigorous physical activity showed the strongest favourable association with QoL. A greater proportion of time spent in moderate-to-vigorous physical activity was linked to higher overall WHOQOL-BREF scores, and the isotemporal substitution results translated this association into interpretable time-reallocation estimates. For example, reallocating 10 min from sedentary behaviour to moderate-to-vigorous physical activity was associated with a 1.49-point higher predicted overall score, while a 30-min reallocation was associated with a 3.87-point higher score. Similar favourable estimates were observed when time was reallocated from sleep or light physical activity to moderate-to-vigorous physical activity, whereas replacing moderate-to-vigorous physical activity with sedentary behaviour showed the most unfavourable predicted difference. These results align with general adult evidence that physical activity is positively associated with QoL (Bize et al., 2007), and with time-use epidemiology studies showing that specific reallocations between movement behaviours can be meaningfully related to mental health and wellbeing outcomes (Dumuid et al., 2019; Kitano et al., 2020). For caregivers of children with intellectual disabilities, moderate-to-vigorous physical activity may represent not only movement intensity but also protected personal time, perceived control, stress relief, and temporary distance from continuous responsibility. At the same time, this finding should be interpreted cautiously. Caregivers who accumulate more moderate-to-vigorous physical activity may also have better baseline health, more flexible employment, safer neighbourhoods, more family support or better access to respite. Thus, the practical implication is not simply to advise caregivers to “exercise more”, but rather to create feasible conditions in which short bouts of higher-intensity movement can occur without displacing necessary sleep, employment or caregiving responsibilities.

The null findings for sleep and light physical activity, considered alongside the log-ratio variation matrix, help clarify the overall pattern. Sleep and sedentary behaviour showed the strongest proportional co-dependence, suggesting that these movement behaviours may be organised by shared routines such as evening supervision, fatigue, night-time vigilance, and household responsibilities. Yet sleep duration was not a statistically clear correlate of overall QoL. One explanation is that duration alone may not capture the aspects of sleep most relevant to caregivers, including sleep fragmentation, night awakenings, anticipatory worry, and perceived restfulness. Prior literature has linked maternal stress, wellbeing, and impaired sleep among mothers of children with developmental disabilities, indicating that sleep quality may be as important as sleep quantity in this group (Lee, 2013). Light physical activity also showed no clear independent association, which is plausible because light physical activity in caregiving households may have mixed meanings: it can include restorative walking, but also housework, errands, and care tasks performed under time pressure. The large variation involving moderate-to-vigorous physical activity, especially relative to light physical activity, suggests that higher-intensity movement is not merely a continuation of routine daily tasks but may require a distinct opportunity. These findings support the value of compositional methods in caregiver research because they make explicit the trade-offs within a finite day (Chastin et al., 2015; Dumuid et al., 2020; Pedišić, 2014). Future studies should incorporate sleep quality, activity context, caregiving timing, respite access, and longitudinal designs to clarify whether changing daily time composition can improve caregivers' own QoL. For now, the results support family-centred strategies that reduce avoidable sedentary time and protect realistic opportunities for caregivers' self-directed movement within the constraints of caregiving life (Vanderkerken et al., 2019).

### 4.1 Strengths and limitations

This study has several notable strengths. First, the application of a 24-h compositional framework accounted for the finite and co-dependent nature of daily time. Second, the objective assessment of caregivers' 24-h movement behaviours using waist-worn accelerometers helped reduce the recall bias inherent in self-reported time-use measures. Finally, the study translated log-ratio regression coefficients into 10- and 30-min reallocation estimates, making the findings more tangible and interpretable for family-centred services and intervention planning.

Nevertheless, several limitations should be considered. The cross-sectional design precludes causal inference and cannot rule out reverse causality. Recruitment through special education schools in a single Chinese city, the predominantly female caregiver sample, and the inclusion of only caregivers of children with moderate or severe intellectual disabilities may limit generalizability to other regions, caregiver groups, and families of children with different levels of intellectual disabilities. Although accelerometers objectively estimated movement behaviour duration, they did not capture activity context, timing, perceived exertion, or sleep quality. Additionally, the analysis focused on the overall WHOQOL-BREF score; domain-specific models may reveal different associations for physical, psychological, social, and environmental QoL. Finally, unmeasured factors such as caregiver depression, child behavioural problems, respite availability, informal support, and neighbourhood safety may confound the observed associations.

## 5 Conclusion

Caregivers' 24-h movement behaviour composition was associated with their own QoL in families of children with intellectual disabilities. The most favourable predicted contrast was the reallocation of sedentary time to moderate-to-vigorous physical activity. These findings support further investigation of family-centred strategies to reduce avoidable sedentary time and facilitate feasible opportunities for physical activity without compromising sleep, employment, essential care, or recovery.

## CRediT authorship contribution statement

**Yanan Liu:** Writing – original draft, Investigation. **Yangchen Liu:** Writing – original draft, Investigation. **Jianing Ding:** Formal analysis, Data curation. **Yaqing Yuan:** Writing – review & editing, Writing – original draft, Methodology, Conceptualization. **Glenn Roswal:** Writing – review & editing.

## Declaration of competing interest

The authors declare that they have no known competing financial interests or personal relationships that could have appeared to influence the work reported in this paper.

## Data Availability

The analytic data are available from the corresponding author on reasonable request.
